# Effects of whole-body vibration training on cognitive function: A systematic review

**DOI:** 10.3389/fnhum.2023.854515

**Published:** 2023-02-09

**Authors:** Jiayi Wen, Lu Leng, Min Hu, Xiaohui Hou, Junhao Huang

**Affiliations:** ^1^Guangdong Provincial Key Laboratory of Physical Activity and Health Promotion, Scientific Research Center, Guangzhou Sport University, Guangzhou, Guangdong, China; ^2^School of Foreign Languages, Jinan University, Guangzhou, Guangdong, China

**Keywords:** whole-body vibration, cognitive function, rehabilitation, cognitive ability, vibration training

## Abstract

**Background:**

Whole-body vibration (WBV) training is a novel training method that stimulates the human neuromuscular system by the use of vibration, the frequency and amplitude of which are controlled, thereby inducing adaptive changes in the body. WBV training is widely used as a clinical prevention and rehabilitation tool in physical medicine and neuro-rehabilitation as a clinical prevention and rehabilitation tool.

**Objectives:**

The aim of the present study was to review the effects of WBV on cognitive function, provide an evidence-based foundation for future research on WBV training, and promote additional popularization and use of the methodology in clinical practice.

**Methods:**

A systematic review of articles extracted from the following six databases was conducted: PubMed, Web of Science, China National Knowledge Infrastructure, Embase, Cochrane, and Scopus. A literature search was performed on articles in which the effects of WBV on cognitive function were evaluated.

**Results:**

Initially, a total of 340 studies were initially identified, among which 18 articles that satisfied the inclusion criteria were selected for inclusion in the systematic review. Participants were allocated into two groups: patients with cognitive impairment and healthy individuals. The results demonstrated that WBV was both positive and ineffective in its influence on cognitive function.

**Conclusion:**

The majority of studies suggested that WBV may be a useful strategy for the management of cognitive impairment and should be considered for inclusion in rehabilitation programs. However, the impact of WBV on cognition requires additional, larger, and adequately powered studies.

**Systematic review registration:**

https://www.crd.york.ac.uk/PROSPERO/display_record.php?RecordID=376821, identifier CRD42022376821.

## Introduction

Whole-body vibration (WBV) training is a method of neuromuscular training which produces oscillations that are transferred to the body and perceived by the muscular-skeletal apparatus (Kim and Lee, 2018; Fereydounnia and Shadmehr, 2020). At the end of the 19th century, this method was first used by Goetz (2009) to treat gait disorders in patients with neurological disabilities, especially those with Parkinson’s disease. WBV may be conducted when a subject either stands stationary (passive exercise) or performs movements while standing, sitting or lying on the vibration platform (active exercise). In the study by Stania et al. (2016) and Pang et al. (2013), subjects performed specific exercise training on the vibration platform, which was considered active WBV training. However, in the articles highlighted in the current review, WBV training was conducted by asking subjects to sit on a bench on a vibrating platform or stand directly on the platform. In the study by Amonette et al. (2015), a static squatting position was adopted using 45° of knee flexion. Here, the body was moving (with reflexive muscle contractions) without active intervention, which was considered a form of passive physical exercise (Fereydounnia and Shadmehr, 2020). Moreover, the mechanical load caused was extremely limited, and was considered safe, allowed for ready adjustment, and ease of use. Therefore, it was general recommended for patients who were weak, untrained, or had defective balance problems.

Cognitive functioning refers to the ability of the human brain to process, store and extract information, that is, the capability to process the occurrence and development of a series of events (Naito et al., 2000). Cognitive functioning includes many independent areas, such as memory, attention, executive ability, feeling, perception, thinking, learning, and judgment. When one or more of the above mentioned functions are impaired, the patient is considered to suffer from cognitive impairment.

After detailed observation, it was concluded that exercise can improve functional activity in the prefrontal cortex, the superior cortex of the central axis, and the marginal cortex. Overall, exercise can improve cognitive functioning (Kingwell, 2019). In accordance with the specific principles of exercise, the benefits of different methods are not equivalent and the relationship between different styles, such as aerobic and resistance exercise, with cognitive capability has been confirmed in previous studies (Karssemeijer et al., 2017; Herold et al., 2019; Moriarty et al., 2019; Stern et al., 2019). However, few studies have been published that have evaluated the relationship between WBV and cognitive function. In 2014, to ascertain the effects of WBV therapy on cognitive functioning in healthy young individuals, Regterschot et al. (2014) treated participants by performing WBV training and concluded that WBV training had a positive short-term effect on executive function in these adults. In Rosado et al. (2021) conducted a randomized controlled trial and found that psychomotor intervention combined with WBV training was effective in preventing falls, cognitive function and physical function decline. However, Santin-Medeiros et al. (2017) believed that 8 months of WBV training in elderly women did not improve the cognitive status. Lam et al. (2018) proposed that WBV training combined with a routine activity program had no significant effect on the cognitive ability of patients with mild or moderate dementia. Furthermore, no systematic reviews are available that have established an association between WBV training and cognitive function. In addition, a clear consensus regarding vibration exposure parameters (i.e., frequency, amplitude, or duration) has not been reached. Hence, the purpose of this systematic review was to review the available literature and critically observe the effect of WBV training on cognitive function.

## Materials and methods

### Search strategy

PubMed, Web of Science, China National Knowledge Infrastructure (CNKI), Embase, Cochrane, and Scopus databases were used to comprehensively and systematically search the literature for articles published prior to December 2021, with no limit on the earliest date. According to the PICO policy, keywords including [“whole-body vibration” or “vibration training” (Title/Abstract)] AND [“cognitive function” or “cognitive control” or “cognitive ability” or “cognition” (Title/Abstract)] were used. The search was limited to full original articles that focused on human subjects without restrictions in the language of publication. The manuscript adheres to the PRISMA guidelines for reporting systematic reviews (Page et al., 2021).

### Inclusion and exclusion criteria

Articles that met the following criteria were selected: meta-analyses, systematic reviews, or experimental research related to WBV training, at least one outcome of the study was related to cognitive function.

Articles were excluded if any of the following exclusion criteria applied: WBV training studies not in a sports or medical field, such as agriculture, construction, transportation, and mechanics; studies focused on the detrimental effects of mechanical vibration in the work environment, for example, when operating tools (e.g., sledgehammers or forming machines) or while riding vehicles (e.g., trucks, helicopters, or tanks); an abstract or conference paper; studies in which WBV was not utilized; participants were not human, for example, animal studies.

### Data collection and analysis

To assess the effects of WBV training on cognitive function and record the principal characteristics of each study, a standardized data extraction and evaluation form developed by the authors was used to record relevant data. Characteristics of the studies included first author, publication year, target population, number of participants, intervention and control groups, outcomes, and WBV specifications. In accordance with the guidelines of van Heuvelen et al. (2021), the WBV specifications included the type of vibration, frequency, amplitude of WBV, duration, and posture. Details of the data extraction process are displayed in Table 1.

**TABLE 1:** Effects of whole-body vibration (WBV) training on cognitive function in humans.

References	Participants	Groups	Frequency and amplitude of WBV	Duration of WBV	Outcome measures	Outcomes
Fereydounnia and Shadmehr, 2020	15 women with normal lordosis and 15 women with lumbar hyper-lordosis	Experimental group: women with lumbar hyper-lordosis (*n* = 15) Control: women with normal lordosis (*n* = 15)	Frequency: 30 Hz, high range: 5 mm	5 times (1 min each)	SART test system	WBV had positive immediate effects on the reaction time in both groups, but it had negative effects on anticipatory skill with high speed in women with normal lumbar lordosis.
Kim and Lee, 2018	18 senile women with suspected mild dementia	Experimental group: WBV (*n* = 9) Control group: no vibration (*n* = 9)	Frequency: 20–40 Hz, amplitude: 3 mm	5 days/week, 8 weeks	EEG, MMSE	WBV training activated the cerebrovascular circulation, having a positive impact on cognitive functioning.
Regterschot et al., 2014	133 healthy participants (112 females, 21 males)	Treatment: WBV (*n* = 133)	Frequency: 30 Hz, amplitude: 0.5 mm	6 times (2 min each)	CBT, CWIT, SDS, DSBT	WBV had a short-term positive effect on executive function (attention and inhibition) in young people.
Rosado et al., 2021	51 participants (aged 75.4 ± 5.6 years)	EG1: psychomotor intervention program (*n* = 16) EG2: psychomotor intervention program + WBV (*n* = 16) Control: daily activities(*n* = 19)	Frequency: 12.6–15 Hz, amplitude: 3 mm	3 times/week (3–6 min each), 24 weeks	CogTUG	Psychomotor intervention combined with WBV training is effective in preventing falls, cognitive function and physical function decline.
Santin-Medeiros et al., 2017	37 women	Treatment: WBV (*n* = 19) Control: no vibration (*n* = 18)	Frequency: 20 Hz, amplitude: 2 mm	2 times/week (30–35 min each), 8 months	Abbreviated mental test	WBV training did not improve HRQoL scores, life satisfaction, cognitive status or fall risk in elderly women.
Lam et al., 2018	54 elderly adults (40 women) with mild or moderate dementia	Experimental group: WBV (*n* = 27) Control group: usual routine (*n* = 27)	Frequency: 30 Hz, amplitude: 2 mm	2 times/week, 9 weeks	CMMSE	No significant difference in CMMSE score or changes in outcomes measured at post-training and at 3-month follow-up identified.
den Heijer et al., 2015	55 healthy children (aged 8–13)	Treatment: WBV (*n* = 55)	Frequency: 30 Hz, amplitude: 0.44–0.6 mm	3 min	The Stroop Color-Word Interference Test	WBV training improved the inhibitory function of children, with a therapeutic effect related to intelligence and age, but not to ADHD.
Amonette et al., 2015	12 healthy participants (8 men and 4 women)	VV: vertical vibration (*n* = 12) RV: rotational vibration (*n* = 12) Control: placebo (*n* = 12)	Frequency: 30 Hz, amplitude: 4 mm	5 times (2 min each)	ImPACT	An acute bout of static squats with a 45° angle of knee flexion accompanied by WBV did not affect visual or verbal memory, reaction time, or impulse control measured using ImPACT, but motor processing speed may have been increased after vertical vibration.
de Bruin et al., 2020	Seventeen elderly adults (10 women, 7 men)	Treatment: WBV (*n* = 9) Control: placebo (*n* = 8)	Frequency: various, amplitude: 3 mm	5 times (1 min each), 3 days/week, 8 weeks	TMT-A, TMT-B	8-week SR-WBV combined with EXDT intervention had a positive effect on physical function and cognition of the care-dependent elderly.
Dennis et al., 2008	A 25-year-old patient with ADHD and 6 healthy college students	Treatment: WBV (a 25-year-old patient with ADHD) (*n* = 1) Control: no vibration (6 healthy college students) (*n* = 6)	Frequency: 30 Hz, amplitude: 0.44–0.66 mm	10 consecutive days, 3 times/day (15 min each)	TAP, Digit Span Backward task, Stroop Color-Word Interference task, controlled oral word association test, items drawn from the attention questionnaire	Both ADHD patient and healthy individuals showed significant improvement in attention, memory, and divergent thinking.
Cabeza et al., 2004	17 patients with ADHD and 83 healthy individuals	Treatment: WBV (*n* = 100)	Frequency: 30 Hz, amplitude: 0.44–0.66 mm	2 min	the Stroop Color-Word Interference task	Both ADHD patients and healthy individuals showed significant improvements in attention.
Yang et al., 2016	25 adults with MS	Treatment: WBV (*n* = 25)	Frequency: 20 Hz, amplitude: 1.3 mm	5 times/day (1 min each), 3 days/week, 8 weeks	PASAT-3″	Cognitive functioning in MS patients was enhanced.
Uhm and Yang, 2018	30 patients with stroke diagnosed within 3 months	Group I: WBV + BPCT (*n* = 10) Group II: AS + BPCT (*n* = 10) Group III: BPCT (*n* = 10)	NR	8 weeks	EEG	WBV combined with computerized postural control training improved muscle and cerebral cortex activity in stroke patients.
Durgut et al., 2020	30 children (7–11 years of age) with ADHD	Group I: TT (*n* = 15) Group II: TT + WBV (*n* = 15)	Frequency: 50 Hz, amplitude: 0–5 mm	3 days/week, 8 weeks (15 min each)	STP-TBAG, BRIEF	TT + WBWT training improved the scores of Stroop test, BRIEF, CRS, PedsQL, and TBAG Form.
Odano et al., 2022	16 patients with aMCI (aged 63.5 ± 8.2 years), 7 men and 9 women	Treatment: WBV (*n* = 16)	Frequency: 35–40 Hz, amplitude: NR	2 times/week (20 min each), 24 weeks	rCBF	WBV exercise and training increase rCBF in aMCI patients, and WBV training enhances cognitive function and may increase the cognitive reserve.
Zhu, 2016	90 male patients with myasthenia	TC: tai chi (*n* = 24) WBV: WBV (*n* = 28) Control: no WBV (*n* = 27)	Frequency: 12–16 Hz, amplitude: 3–5 mm	5 times/day (1 min each), 5 days/week, 8 weeks	MMSE	WBV training had no effect on the MMSE score of cognitive ability compared with the control group.
Boerema et al., 2018	34 humans randomly assigned to a WBV or control group	Treatment: WBV (*n* = 18) Control: no vibration (*n* = 16)	Frequency: 30 Hz, amplitude: 0.5–1 mm	Humans: 4 days/week (4 min each), 5 weeks	The Stroop Test, digit memory span forward/backward, TMT	Cognitive tests in humans revealed a selective improvement in the Stroop Color-Word test after WBV training.
Choi and Mizukami, 2020	24 participants (aged 88.0 ± 5.0 years)	Treatment: SWV (*n* = 13) Control: no SWV (*n* = 11)	NR	5 days/week, 2 months (10 min each)	MMSE, NIRS	The score of MMSE in SWV group was improved, and the brain NIRS also showed that the concentration of oxidized hemoglobin and total hemoglobin increased significantly.

ADHD, attention deficit hyperactivity disorder; aMCI, amnestic mild cognitive impairment; AS, aero-step; BPCT, computerized postural control training; BRIEF, Behavior Rating Inventory of Executive Function; CBT, the Stroop Color-Block Test; CMMSE, Cantonese Mini-Mental State Examination; CogTUG, cognitive TUG test; CRS, Conners’ rating scale; CWIT, Stroop Color-Word Interference Test; DSBT, Digit Span Backward task; HRQoL, health-related quality of life; ImPACT, immediate postconcussion assessment and cognitive test; MMSE, Mini-Mental State Examination; MS, multiple sclerosis; NR, not reported; PASAT-3″, Paced Auditory Serial Addition Test-3 Seconds; PedsQL, pediatric quality of life inventory; rCBF, regional cerebral blood flow; SART, speed anticipation reaction time; SDS, stroop difference score; SWV, Sonic Wave Vibration; STP-TBAG, Stroop Test TBAG form; TAP, test battery of attentional performance; TMT, trail making test; TT, treadmill training; WBVT, whole body vibration training.

**FIGURE 1:**
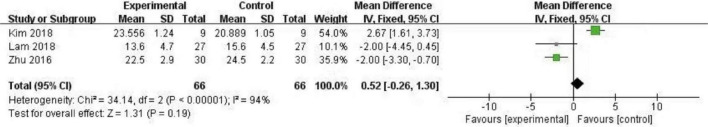
Forest plot of MMSE scale.

**FIGURE 2:**
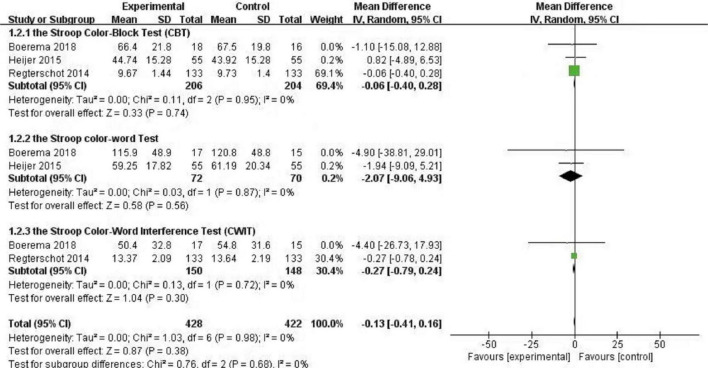
Forest plot of the Stroop Test.

For quantitative analysis, it was found that different studies used different methods to evaluate the data. Four articles used the MMSE scale to measure the main results, whereas six articles used the Stroop Test. The MMSE scale and the Stroop Test are suitable for the use of Review Manager version 5.4 for quantitative analysis. Figure 1 shows forest map of the effect estimation and comparison of the MMSE scale. Figure 2 shows forest map of the effect estimation and comparison of the Stroop Test.

### Quality assessment

The methodological quality of the included articles was assessed by two independent raters using the standardized and validated Physiotherapy Evidence Database (PEDro) scale for quality. The PEDro scale was used to evaluate the scientific rigor of the selected clinical trials (9–10 = excellent, 6–8 = good, 4–5 = fair, and ≤4 = poor) (Centre for Evidence-Based Physiotherapy, 2015). The PEDro scale is an 11-item scale that has previously been used in systematic reviews (Cashin and McAuley, 2020). The results of the assessment of the PEDro scale are presented in Table 2.

**TABLE 2:** Study quality using the PEDro scale.

References	1*	2	3	4	5	6	7	8	9	10	11	Total
den Heijer et al., 2015	1			1				1		1	1	4
Cabeza et al., 2004	1			1				1	1	1	1	5
de Bruin et al., 2020	1			1				1		1	1	4
Yang et al., 2016	1			1				1		1	1	4
Kim and Lee, 2018	1			1				1	1	1	1	5
Uhm and Yang, 2018	1	1		1	1			1	1	1	1	7
Durgut et al., 2020	1	1	1	1	1	1	1	1	1	1	1	10
Lam et al., 2018	1	1		1	1	1	1	1	1	1	1	9
Zhu, 2016	1	1		1	1			1	1	1	1	7
Regterschot et al., 2014	1			1				1		1	1	4
Choi and Mizukami, 2020	1	1		1	1			1	1	1	1	7
Amonette et al., 2015	1	1		1				1	1	1	1	6
Fereydounnia and Shadmehr, 2020	1			1				1	1	1	1	5
de Bruin et al., 2020	1	1		1	1	1	1	1	1	1	1	9
Santin-Medeiros et al., 2017	1	1		1				1	1	1	1	6
Boerema et al., 2018	1	1		1	1	1	1	1	1	1	1	9
Rosado et al., 2021	1	1		1	1			1	1	1	1	7
Odano et al., 2022	1			1				1		1	1	4

1, eligibility criteria and source of participants; 2, random allocation; 3, concealed allocation; 4, baseline comparability; 5, blinded participants; 6, blinded therapists; 7, blind assessors; 8, adequate follow-up; 9, intention-to-treat analysis; 10, between-group comparisons; 11, point estimates and variability.

*Item 1 does not contribute to the total score.

The risk of bias was assessed by collaboration of the two reviewers, and disagreements were resolved by discussion. To evaluate the methodological quality, the criteria of the Cochrane risk of bias tool were used.

### Registration and protocol

Systematic review was performed using the preferred reporting items for the PRISMA checklist. This review was registered in the PROSPERO database (Registration Number: CRD42022376821).

## Results

### Study selection

**FIGURE 3:**
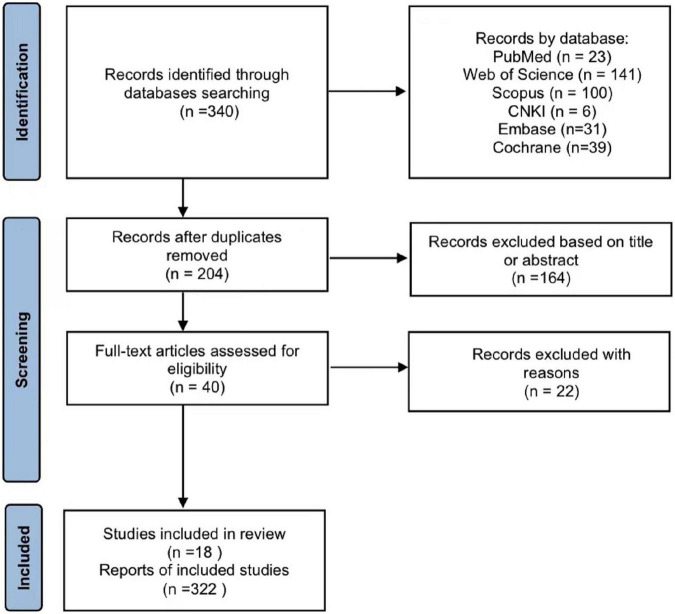
Flowchart representing the process of article selection.

After searching the electronic databases PubMed, Scopus, CNKI, Embase, Cochrane, and Web of Science, 340 potential relevant studies were identified. After removing duplicates, 147 articles remained. In accordance with the inclusion and exclusion criteria, 204 articles were excluded, mostly because the focus was on vibration in agriculture, construction, transportation, and machinery, rather than WBV training related to the sports or medical field. After reading the abstracts, followed by the full text of the articles, 18 studies ultimately satisfied the inclusion criteria and were included in the systematic review. The process for literature screening is displayed in Figure 3. The results of the Pedro scale are presented in Table 2. Articles were managed using Endnote software.

**FIGURE 4:**
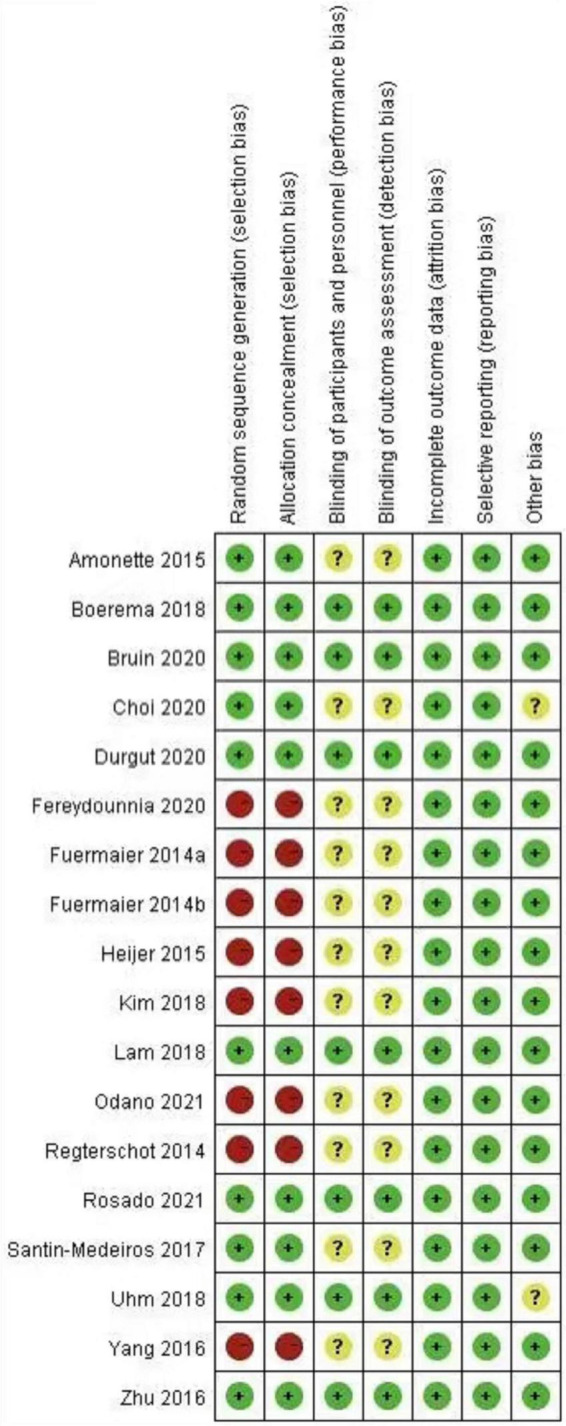
Risk of bias summary.

All 18 articles were thoroughly analyzed and approved by two reviewers using the PEDro scale. Figure 4 shows the risk of bias for each of the included studies. Only one study used a randomized tool for sealing envelopes, and 10 studies mentioned random sequence generation, but did not include information regarding blindness of assessors.

The GRADE was used to assess the certainty of evidence. After completion of the classification of evidence, a summary of evidence and results is presented in Table 3.

**TABLE 3:** Evidence summary form of the GRADE.

Certainty assessment							Number of patients		Effect		Certainty	Importance
Number of studies	Study design	Risk of bias	Inconsistency	Indirectness	Imprecision	Other considerations	Whole-body vibration training	No whole-body vibration training	Relative (95% CI)	Absolute (95% CI)		
**MMSE scale**												
3	Randomized trials	Serious^a^	Not serious	Not serious	Not serious	None	66	66	–	MD **0.52 higher** (0.26 lower to 1.3 higher)	⊕⊕⊕⁣○ Moderate	CRITICAL
**The Stroop Color-Block Test (CBT)**												
4	Observational studies	Very serious^b^	Not serious	Not serious	Not serious	None	207	210	–	MD **0.06 lower** (0.4 lower to 0.28 higher)	⊕⁣○⁣○⁣○ Very low	IMPORTANT
**The Stroop Color-Word Test**												
2	Randomized trials	Serious^c^	Not serious	Not serious	Not serious	None	72	70	–	MD **2.07 lower** (9.06 lower to 4.93 higher)	⊕⊕⊕⁣○ Moderate	IMPORTANT
**The Stroop Color-Word Interference Test (CWIT)**												
3	Observational studies	Serious^c^	Not serious	Not serious	Not serious	None	151	154	–	MD **0.27 lower** (0.79 lower to 0.24 higher)	⊕⁣○⁣○⁣○ Very low	IMPORTANT

CI, confidence interval; MD, mean difference; MMSE, Mini-Mental State Examination; CBT, Color-Block Test; CWIT, Color-Word Interference Test. ^a^Two articles showed that there was no significant change in the MMSE score of cognitive ability after WBV training.

However, one article showed that there was significant change in the MMSE score of cognitive ability after WBV training. ^b^The two articles were observational studies, which were not randomly grouped and not allocated or hidden. An article is a random assignment experiment, and allocation hiding was carried out. ^c^One article was observational experiments, which was not randomly grouped and not allocated and hidden. An article is a random assignment experiment, and allocation hiding was carried out. GRADE Working Group grades of evidence. High certainty: We are very confident that the true effect lies close to that of the estimate of the effect. Moderate certainty: We are moderately confident in the effect estimate: The true effect is likely to be close to the estimate of the effect, but there is a possibility that it is substantially different. Low certainty: Our confidence in the effect estimate is limited: The true effect may be substantially different from the estimate of the effect. Very low certainty: We have very little confidence in the effect estimate: The true effect is likely to be substantially different from the estimate of effect.

### Effect of WBV on the cognitive ability of individuals with normal cognition

Cognition refers to the process of acquisition, coding, operation, extraction, and the use of sensory input information, a psychological process between input and output, including perception, attention, memory, and thinking. The cognitive level in different social groups differs. Therefore, studies have been conducted on healthy individuals or those with sub-optimal health but with normal cognition, such as healthy children, elderly women, and women with lumbar hyper-lordosis.

To ascertain the effects of WBV therapy on cognitive functioning in healthy young individuals, in Regterschot et al. (2014) recruited 133 healthy youths (112 females, 21 males) with a mean age of 20.5 ± 2.2 years to undergo WBV treatment at 30 Hz with an amplitude of approximately 0.5 mm for 2 min, 6 times. The results indicated that Stroop Color-Word Interference Test (CWIT) scores improved, but Digit Span Backward task (DSBT) scores remained unchanged. Finally, it was concluded that 2 min of passive WBV training had a positive short-term effect on executive function (attention and inhibition) in young adults. Subsequently, den Heijer et al. (2015) found showed that 3 min of WBV training at 30 Hz with an amplitude of 0.44–0.6 mm improved the inhibitory function of healthy children, with a therapeutic effect related to intelligence and age, but not to attention deficit hyperactivity disorder (ADHD). In the same year, Amonette et al. (2015) recruited 12 healthy subjects for WBV exercise at 30 Hz with a 4-mm amplitude (2 min each, 5 times) to determine whether WBV exercise reduced neuro-cognition in healthy subjects. The results indicated that WBV training with knee flexion at a 45° angle did not affect visual or verbal memory, reaction time, or impulse control measured using the Immediate Post-concussion Assessment and Cognitive Test (ImPACT), although motor processing speed may increase following vertical vibration. It is worth noting that the study performed by Amonette et al. (2015) emphasized body posture during WBV, with static squats using a squatting angle of 45°. The present review demonstrated an association between slow psychomotor reaction time and lower back pain, with a correlation between lumbar hyper-lordosis and lower back pain. In neurocognitive tests, the choice of reaction time represents an important reference index. In Fereydounnia and Shadmehr (2020) conducted passive WBV training (frequency = 30 Hz, amplitude = 5 mm, duration = 5 min) training on 15 women with normal lumbar lordosis and 15 women with hyper-lordosis of the lumbar spine. The data demonstrated that WBV training had an immediate positive effect on reaction time in both groups, but a negative effect on anticipatory skills with high speed in women with normal lumbar lordosis.

As age increases, physiological functioning in humans gradually declines, and is combined with changes in cognitive functioning. Brain imaging studies have suggested that brain volume changes faster in adults over the age of 50, with an annual decline of 0.35% compared with 0.12% in young individuals. Brain volume has been confirmed to positively correlate with cognitive function (Cabeza et al., 2004; Dennis et al., 2008). In de Bruin et al. (2020) randomly divided 17 elderly individuals into two groups: an intervention group (*n* = 9) and a sham operation group (*n* = 8). The intervention group received 4 weeks of WBV training (1 min each, 5 times, 3 days/week). From weeks 5 to 8, a passive trampoline program of 5 min was introduced following the vibration sessions. The results indicated that the 8-week training program, consisting of a combination of stochastic resonance WBV and exergame-dance training, was beneficial to both physical and cognitive performance in older care home-dwelling adults. In the same year, to clarify the effect of Sonic Wave Vibration (SWV) on cognitive function and autonomic nervous function, Choi and Mizukami (2020) randomly divided 24 elderly patients into a SWV group and a control group. The SWV group received SWV for 10 min a day, 5 days a week for a total of 2 months. Compared with the control group, the MMSE score in the SWV group improved. In addition, the results of brain NIRS showed that the concentration of oxidized hemoglobin and total hemoglobin increased significantly, thereby suggesting that activation of frontal lobe function may be improved. Similarly, in Rosado et al. (2021) conducted a randomized controlled trial on 51 subjects with a mean age of 75.4 ± 5.6 years. Fifty-one participants were allocated into two experimental groups and a control group: EG1 was enrolled in a psychomotor intervention program, EG2 was enrolled in a combined exercise program (psychomotor intervention program and whole-body vibration program), and the control group maintained their usual daily activities. The vibration amplitude (mm) was always 3 and the frequency (Hz) increased from 12.6 to 15. Rosado et al. (2021) indicated that psychomotor intervention combined with WBV training was effective in preventing falls, cognitive function and physical function decline. However, Santin-Medeiros et al. (2017) did not concluded this. A total of 37 elderly women, with a mean age of 82.4 ± 5.7 years was randomly divided into two groups: vibration (*n* = 19) and control (*n* = 18) groups. WBV training for 8 months at 20 Hz with a 2-mm amplitude (30–35 min each, twice per week) did not improve health-related quality of life (HRQoL) scores, life satisfaction, cognitive status, or fall risk in elderly women. Inconsistencies in related research studies may be due to differences in the evaluation and testing methods. Santin-Medeiros et al. (2017) examined cognitive capability using intelligence tests. Both Santin-Medeiros and Zhu concluded that WBV training did not improve cognitive function, but there were multiple differences between the two subject types (male patients with myasthenia and elderly women). Few studies that focused on the relationship between WBV and cognition have been published, the results of which are contradictory. The majority of studies have demonstrated that WBV training improved cognitive performance, with only a small number concluding that it does not.

### Effect of WBV on the cognitive ability of patients with cognitive impairment

Cognitive impairment generally refers to problems with memory, attention, the learning of new information, planning, organization, and decision making. The degree of injury closely relates to the type of disease and its duration. Cognitive rehabilitation is a type of therapy that principally aims to improve attention, memory, and executive function (Chung et al., 2013).

The use of WBV training to ameliorate cognitive impairment was first proposed in 2014 when Fuermaier et al. (2014a) studied a 25-year-old patient with ADHD and 6 healthy college students with a mean age of 22.8 ± 2.4 years. It was found that 10 days of WBV treatment at 30 Hz with an amplitude of 0.44–0.66 mm improved attention, memory, and divergent thinking in both ADHD patients and healthy individuals. In the same year, Fuermaier et al. (2014b) continued to conduct in-depth research and verified these results using an increased sample size. A total of 83 healthy individuals aged 18–31 and 17 ADHD patients aged 21–28 underwent acute WBV training at 30 Hz with a 0.44–0.66 mm amplitude. The data showed that 2 min of WBV training improved attention in both healthy individuals and ADHD patients. Executive function refers to a group of cognitive processes involving attention, working memory, and cognitive flexibility, essential for higher-order mental functioning (Logue and Gould, 2014; Regterschot et al., 2014). Subsequently, to determine the effect of 8-weeks of WBV training on the extent of disabilities in patients with MS, Yang et al. (2016) exposed 25 MS patients with a mean age of 50.3 ± 14.1 years to 20 Hz vibrations with a 1.3-mm amplitude (1-min exposure to vibration in each group, 5 groups per day, 3 days per week, with a 1-min rest between groups). Patients were evaluated using a Paced Auditory Serial Addition Test (PASAT-3), a commonly used scale of cognitive scores, that included processing speed when evaluating auditory information, computing ability, continuous attention, and distraction. Yang et al. (2016) demonstrated significant changes in cognitive ability in MS patients. Finally, it was concluded that 8 weeks of controlled WBV training reduced the extent of disability in MS patients. PASAT-3 scores used by Yang et al. were not used in other studies to test the relationship between WBV training and cognition. Instead, a Stroop Test and Mini-Mental State Examination (MMSE) have been widely used in other studies. In Kim and Lee (2018) studied women with senile dementia aged 65 or above, and randomly divided them into vibration (*n* = 9) and control groups (*n* = 9) for 8 weeks of WBV training at 20–40 Hz. Finally, the data indicated that WBV training activated the cerebral cortex, which had a positive impact on cognitive functioning. In terms of cognitive assessment methods, Kim et al. used electroencephalograms (EEG). Uhm and Yang (2018) also observed the effects of 8 weeks of WBV training combined with computerized postural control training on the cognitive ability of 30 stroke patients within 3 months of diagnosis using EEG. The results showed that this mixed training method improved muscle and cerebral cortex activity in stroke patients. The studies performed by Kim et al. and Uhm et al. are the only two studies that used EEG to analyze cognitive function following WBV exercise intervention. To compare the effects of treadmill training (TT) and WBV training on attention, the severity of ADHD symptoms in patients, impairment of executive function behavior, and the quality of life in children with ADHD, Durgut et al. (2020) randomly allocated 30 children with ADHD into two groups: a “TT” group and “WBVT + TT” group. Both groups received TT for 8 weeks (3 days/week), and the “WBVT + TT” group received an additional 8 weeks of WBV training at 50 Hz at a 0–5 mm amplitude. The results demonstrated that TT + WBWT training improved the Stroop Test TBAG (Scientific and Technological Research Council of Turkey) (STP-TBAG) and Behavior Rating Inventory of Executive Function (BRIEF) scores. In recent study, Odano et al. (2022) conducted WBV training (frequency = 35–40 Hz, duration = 20 min) on 16 patients with amnestic mild cognitive impairment (aMCI). The results demonstrated that WBV exercise and training increased rCBF in aMCI patients. Moreover, WBV training enhanced cognitive function and may increase cognitive reserve.

Although the majority of studies demonstrated that WBV training improved the cognitive ability of patients with cognitive impairment, after collation and extraction of data from relevant manuscripts, a small number of studies showed conflicted findings. In Lam et al. (2018) published results that were the converse of these conclusions. They studied the effects of WBV combined with a routine activity program on lower limb strength, balance, and mobility in community-dwelling individuals with mild or moderate dementia (Lam et al., 2018). They also used the Cantonese Mini-Mental State Examination (CMMSE) scale to evaluate the effect of WBV on the cognitive performance of 54 elderly patients with mild to moderate dementia (40 of whom were women). No significant differences in CMMSE scores or changes in outcomes were identified post-training or at the 3-month follow-up. In addition, myasthenia may be an important risk factor for cognitive impairment, since a clear correlation was found between them (Liu et al., 2020). In a clinical randomized controlled study of tai chi and WBV therapy in the elderly published by Zhu (2016), 90 male patients with myasthenia were randomly divided into WBV, tai chi, and control groups (Yaqiong, 2016). The WBV group underwent 12–16 Hz WBV training for 8 weeks (1 min for each group, 5 groups per day, 5 days per week). An MMSE was used to evaluate the cognitive ability of subjects before and after the experiment. The results indicated no significant changes in cognitive ability in the three groups after the experiment. Thus, WBV training did not affect cognitive function in male patients with myasthenia. The results of that study were quite different from the results of other studies, possibly due to differences in amplitude, frequency, and training posture. Therefore, it has been suggested that an in-depth study of amplitude and other factors should be conducted. The differences may also be related to monitoring and evaluation indicators and methods. As science and technology continuously progress, methods of evaluation and studying cognitive function constantly improve. Future studies should combine a variety of methods that perform evaluation not only using scales of intelligence, language, memory, and attention but also using objective methods, such as EEG, functional magnetic resonance imaging, functional near-infrared spectroscopy, and transcranial Doppler ultrasound.

## Discussion

The aim of this systematic review was to determine changes in cognitive ability after WBV training in healthy individuals and in patients with cognitive impairment. The key findings of the majority of studies included in this review were that WBV training had a positive effect on healthy individuals and those with cognitive impairment. Three studies produced contradictory results, thereby suggesting that WBV treatment had no significant effect on the cognitive ability of healthy individuals and patients with cognitive impairment. WBV training provides a potential cognitive rehabilitation technique for patients with weakness or balance defects. No systematic review has been performed comparing WBV training in cognitive improvement. The present systematic review is important because it analyzes WBV training as a potential aid to cognitive rehabilitation.

Until now, the underlying mechanisms of WBV training that improve cognition remained unclear. Literature review over the years has shown that the majority of studies support the following hypotheses: vibrations produced by WBV can stimulate skin mechanosensory receptors, such as tactile corpuscles, and these mechanoreceptor signals are transmitted to the primary somatosensory cortex. The areas that are associated have a direct and indirect connection with the prefrontal cortex, a region strongly involved in cognitive processing (Braak et al., 1996; Regterschot et al., 2014). The indirect pathways involve the limbic system (such as the amygdala and hippocampus, important areas of learning and memory), which can mediate the effects of sensory correlations on the prefrontal cortex (Durgut et al., 2020). Furthermore, the amygdala has projections to non-thalamic nuclei (e.g., the cholinergic nuclei of the basal forebrain) that have diffuse connections to several brain regions (Braak et al., 1996). Therefore, it has been speculated that this sensory stimulation can improve the cognitive function of the brain by affecting neural transmission of the prefrontal cortex and in regions around the inferior frontal sulcus by increasing the connectivity between neuronal dendrites. A number of studies have demonstrated that WBV training can change the neuromuscular recruitment pattern and muscle length, stimulate muscle spindles, and induce a stretch reflex response, which ultimately leads to stimulation of afferent neurons and irritability of the corticospinal pathways, with an increased oxygen uptake and heart rate (Amonette et al., 2015; Lam et al., 2018; Fereydounnia and Shadmehr, 2020). Changes in heart rate after WBV intervention not only depended on the sympathetic and parasympathetic balance but also directly correlated with the level of activity in the prefrontal cortex (Herrero et al., 2011). In addition, Kim and Lee (2018) performed EEG analysis and demonstrated that, after WBV training, alpha waves in the frontal lobe, which can activate the cerebral cortex, increased significantly, and are beneficial for cognition.

Furthermore, when data from the literature were collected and analyzed, it was found that WBV training could improve the cognitive ability of animals. In Keijser et al. (2017) described the relationship between WBV training and cognition in the mouse for the first time using a model of WBV in which attention and motor performance were improved. They randomly divided 44 male mice into a WBV and control group. The WBV group received 5 weeks of WBV training at 30 Hz and 1.9 g amplitude, 5 days a week for 5 or 30 min on each occasion, while the control group received sham vibration training. It was found that short-term WBV training improved the attention and motor performance of mice. Subsequently, Boerema et al. (2018) trained 10 mice at 30 Hz for 5 weeks (10 min per day, 5 days per week), then analyzed them with positron emission tomography. The results indicated that brain glucose uptake did not change, but motor performance improved (Boerema et al., 2018). Finally, combined with a selective improvement in the human Stroop Test, it was concluded that WBV is a safe intervention that improves brain function. However, the data showed that WBV training improved brain function, but specific improvements in brain function were not analyzed. In the same year, Raval et al. (2018) explored the effectiveness of WBV at reducing post-ischemic stroke and brain injury in reproductively senescent female rats. The animals were divided randomly into a WBV and non-WBV group (Raval et al., 2018). The WBV group was treated with WBV at 40 Hz for 30 days (15 min, twice per day, 5 days per week). Motor function and markers of brain inflammation were measured using histopathology, the results demonstrating that compared with the non-WBV group, inflammatory markers and the volume of the infarct decreased significantly, the level of brain-derived neurotrophic factor increased significantly, and functional activity improved significantly in the WBV group. The principal mechanisms by which WBV training activated/increased the cognitive ability of mice are as follows: (I) forebrain cholinergic system activity; (II) glucose transport across the blood-brain barrier; (III) immediate early gene expression (enhancing neuronal responsiveness); (IV) production of proteins required for neuronal plasticity; (V) production of new neurons; (VI) increased concentration of tyrosine hydroxylase, the enzyme responsible for the synthesis of the precursor of the neurotransmitter dopamine (Fuermaier et al., 2014a). Studies have shown that dopamine affects exercise, motivation, and cognition, and it has been confirmed that it is associated with the pathophysiology of ADHD (Fuermaier et al., 2014a).

Despite the positive findings reported in this systematic review, the discrepancies in the literature regarding the benefits of WBV training on cognitive ability require explanation. Improvements in cognition observed with WBV training may depend on a variety of factors that interact with one another, such as frequency, amplitude, and duration of intervention, possibly explaining the contradictory results. Based on the available data, analysis demonstrates that a beneficial frequency of vibration is from 12 to 50 Hz and an amplitude of 0.44 to 5 mm. Eight studies used a vibratory frequency of 30 Hz, but various amplitudes. This may be related to its mechanism of action. The study by Regterschot et al. (2014) suggests that mechanoreceptors in the skin, such as the Meissner corpuscles, are particularly sensitive to vibrations at 30–40 Hz. These differences make it difficult for parameters of an exercise program to be established, and it is not possible to draw a clear conclusion to determine the best parameters for WBV training. In addition, the methods and means of evaluating cognitive function are also important in qualifying the analysis of the research results. It is worth noting that of the 15 articles included in the study, the Stroop Test is the most frequently used method of evaluation of cognitive function. In addition, several studies chose EEG, reaction time, and MMSE to analyze the experimental results. Interestingly, in three articles, the MMSE scale was used to evaluate the effects of WBV training on cognitive function, while in two articles, it was considered that WBV training did not have a positive effect on cognitive function.

## Limitations

The current review has several limitations that should be considered when interpreting the results. Firstly, only a small number of publications related to WBV and cognitive function were found. Secondly, the search strategy did not include a search for any unpublished literature in this area. Therefore, it is possible that relevant studies may have been missed. Thirdly, due to methodological differences in biomechanical parameters, the type of vibration, and variability in the duration of treatment, the conclusions of this review should be interpreted carefully. Finally, the populations included in these studies were heterogeneous due to differences in age and symptoms. In addition, the guidelines of van Heuvelen et al. (2021) are recommended to promote correct, complete, and consistent WBV reporting for future studies.

## Conclusion

To summarize, WBV training is a method that stimulates and promotes the central nervous system by causing repeated contractions and relaxation in muscles through rapid vibration. Its application and related studies in the field of cognition are still considered novel. We have reviewed relevant studies of the role of this technology in cognitive function and found that WBV training has some contradictory effects on cognition and brain function, but the most studies suggest that WBV training positively influences cognitive performance. Although there are individual studies that suggest that WBV treatment does not significantly improve cognitive ability, it has been pointed out that WBV exercise does not negatively affect neuro-cognition. Therefore, WBV training should be considered for inclusion in rehabilitation programs, but further studies are required to strengthen the reported results. There has been no unified or standardized research design or method of intervention, possibly an important reason for differences in the conclusions of each study. We anticipate that this review will promote studies on the relationship between WBV and cognitive function, with further studies and exploration of the dose-effect relationship of WBV, physical parameters of vibration, the therapeutic effects on different subjects, and potential mechanisms of adaptive change in cognitive ability. Finally, this could result in the development of a scientifically based and effective WBV training system.

## Data availability statement

The original contributions presented in this study are included in the article/supplementary material, further inquiries can be directed to the corresponding author.

## Author contributions

JW, JH, and LL drafted the project plan and protocol. JW performed the literature search. JW and LL screened and evaluated the articles. JH and JW performed the statistical analysis. MH performed the evaluation of clinical relevance. MH and XH supported the analysis of WBV training during the screening process. All authors were involved in data interpretation, drafting of the manuscript, and revisions.
